# Determining the Leaf Emissivity of Three Crops by Infrared Thermometry

**DOI:** 10.3390/s150511387

**Published:** 2015-05-15

**Authors:** Chiachung Chen

**Affiliations:** Department of Bio-industrial Mechatronics Engineering, National ChungHsing University, 250 Kuokuang Road, Taichung 40227, Taiwan; E-Mail: ccchen@dragon.nchu.edu.tw; Tel.: +886-4-2285-7562; Fax: +886-4-2285-7135

**Keywords:** infrared thermometer, leaf emissivity, *Phalaenopsis*, *Paphiopedilum*

## Abstract

Plant temperature can provide important physiological information for crop management. Non-contact measurement with an infrared thermometer is useful for detecting leaf temperatures. In this study, a novel technique was developed to measure leaf emissivity using an infrared thermometer with an infrared sensor and a thermocouple wire. The measured values were transformed into true temperatures by calibration equations to improve the measurement accuracy. The relationship between two kinds of measurement temperatures and setting emissivities was derived as a model for calculating of true emissivity. The emissivities of leaves of three crops were calculated by the mathematical equation developed in this study. The mean emissivities were 0.9809, 0.9783, 0.981 and 0.9848 for *Phalaenopsis* mature and new leaves and *Paphiopedilum* and Malabar chestnut leaves, respectively. Emissivity differed significantly between leaves of Malabar chestnut and the two orchids. The range of emissivities determined in this study was similar to that in the literature. The precision of the measurement is acceptable. The method developed in this study is a real-time, *in situ* technique and could be used for agricultural and forestry plants.

## 1. Introduction

Many sensors have been used to monitor the climate and substrate conditions for agricultural production [1]. Leaf temperatures can provide important information about transpiration, heat or water stress and disease conditions. Hatfield [2] used a portable infrared thermometer to measure foliage temperature and calculate a crop water stress index (CWSI). Jones *et al.* [3] reviewed the method of monitoring stomatal closure of grapevines in the field with infrared thermography. Leinonen and Jones [4] measured the temperature distribution of sunlit and shaded leaf area of *Vinifera* canopies, and then compared the effects of irrigation treatments. Peters and Evett [5] detected the canopy temperature and calculated the CWSI and field temperature mapping for three crops and found that irrigation could be scheduled with their method. The drought response of plants was studied by measuring leaf temperature and two other variables [6]; the advantages of the thermal infrared imagery were non-contact use and sampling more leaves. Maes *et al.* [7] defined the stomatal conductance index and calculated it from measured leaf temperature and two other specific temperatures. Durigon and Lier [8] compared the measurement of the canopy temperature and soil tension for prediction of crop water stress and found that plant-based measurements provided direct insight into plant status, therefore, plant temperature measurements are important.

Plant temperatures can be measured by contact or non-contact sensors. In contact sensors, such as thermocouples or thermistors, the sensor element must have good contact with the sampled object [3]. To ensure accurate measurement of plants, the sensor should be inserted into the plant tissues. However, with thin leaves, this insertion is difficult and usually causes damage to the plants. The adherence of the sensor and the plant also limits the sampling. Temperature measurement of plants by infrared thermometers has become popular since the 1960s. Non-contact and non-restrictive techniques are convenient. However, some factors affecting the performance of the infrared thermometer are the background radiation, and setting appropriate values for the emissivity and calibration of the infrared thermometer [2,3,4,5,6].

Infrared temperature is usually calibrated by a blackbody cavity. Amiro *et al.* [9] designed a leaf chamber for the calibration of infrared thermometers. Calibration for the Everest 4000A radiometer was introduced by Rapier and Michael [10] and the polynomial calibration equation was used to improve performance. Bugbee *et al.* [11] found that longer response period was important to reduce measurement errors, and the polynomial equation was an adequate model for calibration. Savage and Heilmal [12] tested twenty-one infrared thermometers and proposed a third-order polynomial equation for calibration. Chen *et al.* [13] evaluated two types of infrared thermocouple thermometers and used three-order polynomial calibration equations for calibration. The accuracy of this infrared thermistor was within 0.4 °C.

The emissivity represents the ratio of the radiant energy emitted by a surface to that emitted by a blackbody at the same temperature [14]. An emissivity value is set to calibrate the absolute temperature of the sampled object. The infrared thermometer has an emissivity adjustment function for users to adjust the emissivity according to the physical properties of the target surface. Selecting the correct numerical value of the emissivity is the key for accurate measurement with the infrared thermometer [9,10,11,12,13].

The principles and method of determining emissivity for vegetables using an infrared thermometer have been proposed [15]. A standard test method, E1933-99a is issued by American Society for testing and materials (ASTM) [16].

Values of emissivities for alfalfa, Sudan grass, snap bean and tobacco were 0.97, 0.98, 0.96 and 0.97, respectively. Hipps [17] used this method and found emissivities for soil and *Artemisia tridentate* of 0.93 and 0.97. Rubio *et al.* [18] developed two types of the box method to determine vegetation emissivity in the 8–14 μm wave-band range. Sugita *et al.* [19] studied the error sources in determining canopy emissivity and suggested an error of 0.01 for emissivity determination. Rahkonen and Jokela [20] measured the emissivities of three *Brassia rqpa* and *Sonchus arrensis* leaves with a reference emittance technique where a well-stirred water bath was used as the reference environment to maintain a 45 °C temperature. The determined emissivity value was 0.98 ± 0.01 for three types of leaves. This technique was modified and a thermographic camera was used to evaluate the radiation emissivity from nine horticultural crop leaves [21]. These mean emissivity values for the upper and lower sides of leaves were all close to 0.98. The emissivities of intertidal macrroalgae were determined by a complex process [22].

Recently, orchids and ornamental crops have become the high economic value crops cultured in greenhouses. The leaf temperature is the important index for the health state of plants [23,24]. The emissivities of these crops need to be determined using an infrared thermometer.

The performance of commercial infrared thermometers has been improved [13,25]. Uncertainty analysis showed that the ambient temperature did not have a significant effect and an adequate calibration equation could significantly improve the accuracy [25]. Recently, two types of temperature sensors—non-contact infrared thermometer and contact thermocouple wire—have been installed in the infrared thermometer. This device can be used to measure leaf temperatures with different sensing techniques on the same object, providing an opportunity to determine the emissivity of materials.

In this study, a simple and novel technique was developed to determine the emissivities of leaves for three horticultural crops using an infrared thermometer. The determined emissivities were then compared with data from the literature.

## 2. Experimental Section

### 2.1. Theoretical Considerations

Measurement of the radiation physics of plants has been reviewed in detail [26]. The total radiation entering an infrared thermometer has three sources: (1) the observed object itself; (2) other objects reflected on the target surface and (3), the transmittance of the energy from other sources [15,21,26]. Because of the optical properties of plant leaves, the effect of the transmittance energy could be neglected. The radiance energy for leaves is then calculated as:

(1) $$R_{T}=\text{εα}{T_{l}}^{4}+(1-\text{ε})\text{α}{T_{ref}}^{4}$$

where $R_{T}$ is the emitted energy at wavelength 6.0–14.0 µm in Wm^−2^, ε is the emissivity of plant leaves, α is the Stefan-Boltzmann constant (5.67 × 10^−8^ Wm^−2^K^−4^), T*_l_* is the leaf temperature in K, and T*_ref_* is the background temperature in K.

The experiments were performed at night and the microclimate of the greenhouse was uniformly maintained with circulating fans. The second part of the Equation (1) was omitted. The radiation energy detected by an infrared thermometer is calculated as:

(2) $$E_{ir}={\text{ε}}_{is}{T_{is}}^{4}+E_{1}$$

where $E_{ir}$ is the radiation energy detected by infrared thermometer, ${\text{ε}}_{is}$ is the set emissivity of the infrared thermometer, $T_{is}$ is the detected temperature by infrared thermometer and $E_{1}$ is the measurement error.

The radiation energy calculated from the temperature detected by the thermocouple is calculated as:

(3) $$E_{tr}={\text{ε}}_{t}{T_{tr}}^{4}$$

where $E_{tr}$ is the calculated radiation energy, ${\text{ε}}_{t}$ is the true emissivity of leaves and $T_{tr}$ is the leaf temperature detected by thermocouple.

Combining Equations (2) and (3):

(4) $${\text{ε}}_{t}{T_{tr}}^{4}={\text{ε}}_{is}{T_{is}}^{4}+E_{1}$$

(5) $${\text{ε}}_{t}={\text{ε}}_{is}{\left(\frac{T_{is}}{T_{tr}}\right)}^{4}+\frac{E_{1}}{{T_{tr}}^{4}}$$

where $E_{1}/{T_{ir}}^{4}$ is the measurement error, assumed to be normally distributed, uncorrelated, and with mean of zero.

If the ${\text{ε}}_{is}$ is correct, no error exists:

(6) $${\text{ε}}_{t}={\text{ε}}_{is}{\left(\frac{T_{is}}{T_{tr}}\right)}^{4}$$

The term of ${\text{ε}}_{is}{\left(\frac{T_{is}}{T_{tr}}\right)}^{4}$ was defined as the ratio. The ratio value was assumed a linear relationship with value ${\text{ε}}_{is}$:

(7) $$Ratio=b_{0}+b_{1}{\text{ε}}_{is}$$

The calculated term ${\text{ε}}_{is}{\left(\frac{T_{is}}{T_{tr}}\right)}^{4}$ is the dependent $y_{i}$ value and ${\text{ε}}_{is}$ is the independent $x_{i}$ for a linear equation. The parameters of $b_{0}$ and $b_{1}$ can be found by the least squares method. The sampling number was more than 33. The measuring errors of infrared thermometer were reduced with this statistical method. If the set value ${\text{ε}}_{is}$ is equal to the actual value of the emissivity of leaves:

(8) $${\text{ε}}_{t}={\text{ε}}_{is}$$

Combining Equations (7) and (8):

(9) $${\text{ε}}_{t}=b_{0}+b_{1}{\text{ε}}_{t}$$

(10) εt=b01−b1

The ${\text{ε}}_{t}$ value can be calculated from the regression parameters of $b_{0}$ and $b_{1}$. From Equation (6):

(11) $$T_{is}={\left(\frac{{\text{ε}}_{t}}{{\text{ε}}_{is}}\right)}^{0.25}T_{tr}$$

If the setting value of ${\text{ε}}_{is}$ is not correct, the error due to the inaccurate setting value of emissivity is calculated as:

(12) $$\mathrm{Error}=T_{is}-T_{tr}$$

### 2.2. Plant Materials

Three horticultural crops used for this study: Two were orchids: *Phalaenopsis* (*Phal.* Taisuco Anna ‘Taisuco K71303’) ( and *Paphiopedium* (*Paph.* Michael Koopowitz ‘Miao Hua’ (*philippinense × sanderianum*), and the other was a popular ornamental crop, *Pachira macrocarpa* (Malabar chestnut). Two kinds of *Phalaenopsis* leaves were used. New leaves represented the un-folded and 1st leaf (computed from the top of the plant). Mature leaves were the 2nd and 3rd leaves.

### 2.3. Infrared Thermometer

The specifications of the infrared thermometer, a Sentron SI20 LBE (Sentron Eng. Co, Taipei, Taiwan) are given in Table 1.

**Table 1:** Specifications of the SI20 LBE infrared thermometer.

Items	Infrared Thermometer	K-Type Thermocouple
Measuring range	−60–76 °C	−60–14 °C
Operating range	0–50 °C	0–50 °C
Accuracy	2.0 °C	1.0 °C
Resolution	0.1 °C	0.1 °C
Response time	0.5 s	1.0 s
Field of view	30:1	
Emissivity range	0.1–1.0	
Adjusted step	0.01	
Signal indication	LCD screen	LCD screen

### 2.4. Standard Temperature

The standard temperature for thermocouple calibration and the black source for the infrared thermometer were maintained by using a TC 2000 temperature calibrator (Instutek As, Larvik, Norway). The temperature of this standard environment was detected by a resistive temperature detect (RTD) thermometer calibrated by the NIST (National Institute of Standards and Technology, Washington, DC, USA). The uncertainty of this standard equipment was 0.03 °C.

### 2.5. Calibration of Thermometers

The target temperature for calibration was maintained at 10, 15, 20, 25, 30, 35 and 40 °C for the infrared thermometer and K-type thermocouple. Calibration procedures for infrared thermometers [13] and thermocouples [27,28] have been discussed in detail. The accuracy after calibrating was 0.15 and 0.35 °C for the K-type thermocouple and infrared thermometer, respectively.

### 2.6. Leaf Emissivity Measurement

The plants were cultivated in a greenhouse. Ventilation was maintained by circulating fans to ensure a uniform temperature distribution in the greenhouse. The experiments were performed during the night period to reduce the effect the sun radiation. The air temperature of the greenhouse was maintained from 20–32 °C. To avoid damage to the plant tissue, the thermocouple wire (0.076 mm) was attached at the surface of the leaf with permeable tape.

First, the emissivity of the infrared thermometer was set at 0.9. The position of the infrared measurement was near the thermocouple wire and indicated by the laser point. The temperature of the infrared thermometer was recorded. The leaf temperature detected by the thermocouple wire was recorded at the same time. The temperature was measured three times.

Second, the emissivity of the infrared thermometry was adjusted to 0.91. The leaf temperature was detected with the infrared thermometer and thermocouple, and then the emissivity of the infrared thermometer was adjusted to 0.92. The temperature was detected sequentially. The emissivity of the infrared thermometer was adjusted from 0.90 to 1.0. The two types of temperatures were recorded and transformed into true temperatures by specific calibration equations. The true temperatures were then analyzed.

### 2.7. Statistical Analysis

The determination emissivities were analyzed. The average emissivity ${\text{ε}}_{ave}$ and its standard deviation *sd* were calculated. The coefficient of variance (CV) was calculated to evaluate the precision of the measurement:

(13) $$\mathrm{CV}={\text{ε}}_{ave}/sd$$

One-way ANOVA was used for comparison, with Tukey’s *post-hoc* test. Statistical analysis involved use of Microsoft Excel 2010. *p* < 0.05 was considered statistically significant.

## 3. Results and Discussion

### 3.1. Performance of the Sentron SI20 LBE Infrared Thermometer

The calibration equations for the Sentron SI20 LBE infrared thermometer from 10 °C to 40 °C are as follows:

(a) Thermocouple

(14) $$y=-0.00086x^{2}+1.055093x-1.69871$$

(b) Infrared thermometer

(15) $$y=-0.00257x^{2}+1.144658x-1.64894$$

### 3.2. Emissivity of Phalaenopsis Leaves

**Figure 1:**
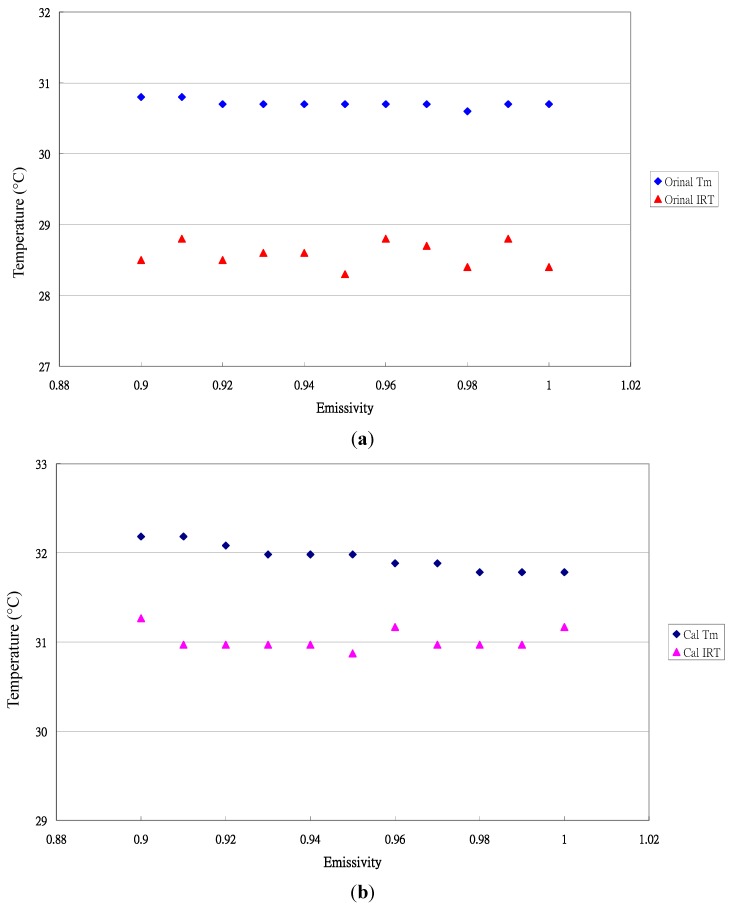
Typical distribution of the original and transformed measured data for *Phalaenopsis* leaves. (**a**) Original temperature data; (**b**) Transformed data.

The typical data distribution for temperature measurement by both sensing elements is shown in Figure 1. The different data distribution patterns between the original measured data (Figure 1a) and the true measured data transformed by calibration equations (Figure 1b) indicate the importance of the calibration equation to obtain accurate data.

**Figure 2:**
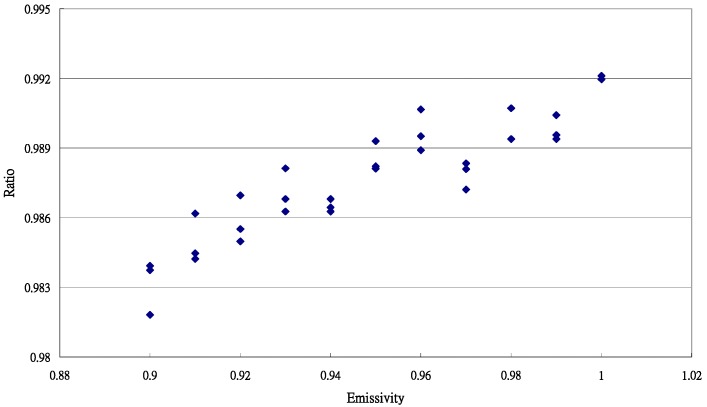
Typical data distribution for the ratio (εisTis4Ttr4) and ${\text{ε}}_{is}$ value for *Phalaenopsis* leaves.

The data distribution for the ratio and ${\text{ε}}_{is}$ value is shown in Figure 2.

The linear equation established by regression analysis with the least squares method is as follows:

(16) $$y=0.073138x+0.918268\;R^{2}=0.8368,s=0.001056$$

The validity of the linear equation was determined by *t* test and residual plots. By the values of $b_{0}(0.918268)$ and $b_{1}$ (0.073138), a typical emissivity of *Phalaenopsis* leaves can be calculated as:

(17) εi=b01−b1=0.918268(1−0.073138)=0.99187

### 3.3. Emissivity of Paphiopedilum Leaves

**Figure 3:**
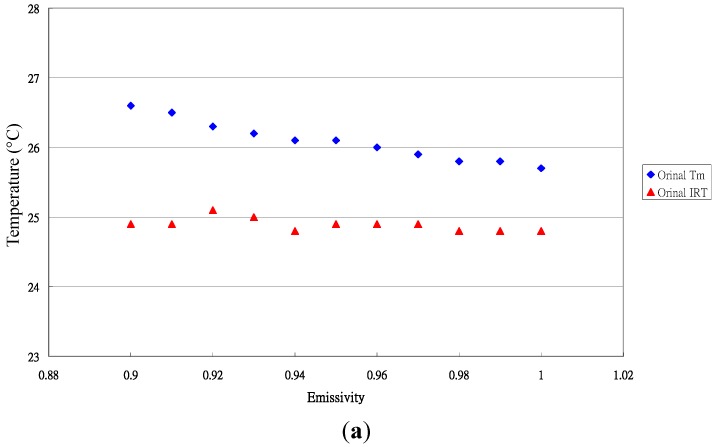

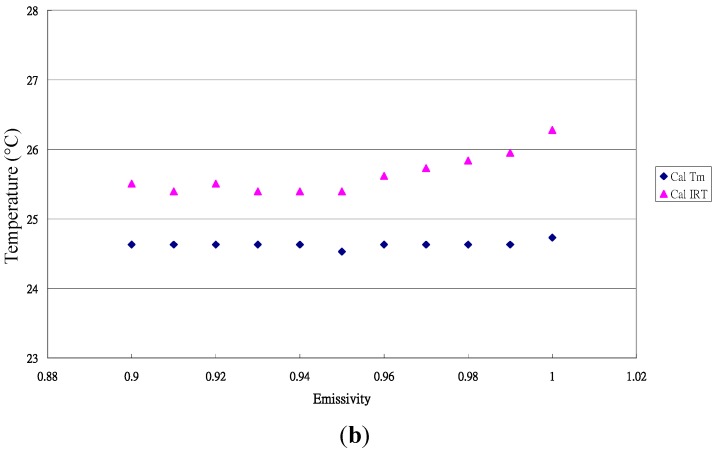
Typical distribution of the original and transformed measured data for *Paphiopedilum* leaves. (**a**) Original temperature data; (**b**) Transformed data.

The typical distribution of the original and transformed temperature for *Paphiopedilum* leaves is shown in Figure 3.

**Figure 4:**
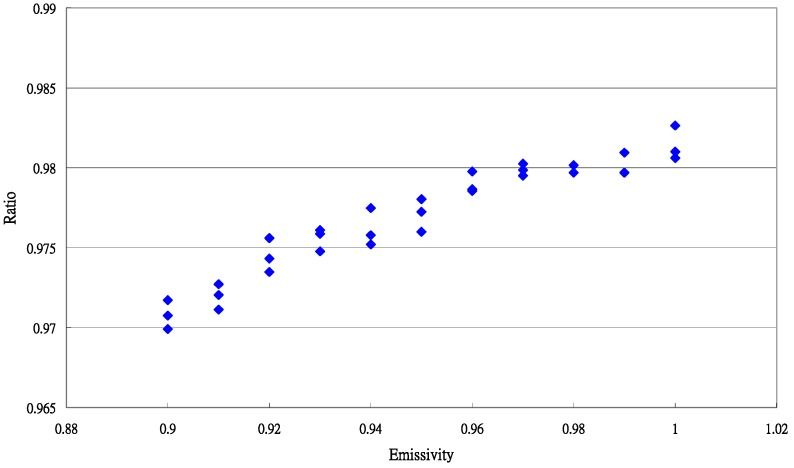
Typical data distribution between for the ratio (εisTis4Ttr4) and ${\text{ε}}_{is}$ value for *Paphiopedilum* leaves.

The accuracy of the measurement was improved by its special calibration equation. The data distribution for the ratio and ${\text{ε}}_{is}$ value is shown in Figure 4.

The result of the regression is as follows:

(18) $$y=0.103493x+0.878635\;R^{2}=0.8945,s=0.001035$$

Using the values of $b_{0}(0.878635)$ and $b_{1}(0.103493)$, the typical emissivity of *Paphiopedilum* leaves was calculated as:

(19) $${\text{ε}}_{i}=\frac{b_{0}}{1-b_{1}}=\frac{0.878635}{1-0.103493}=0.98006$$

### 3.4. Emissivity Variation among Crop Leaves in This Study

**Figure 5:**
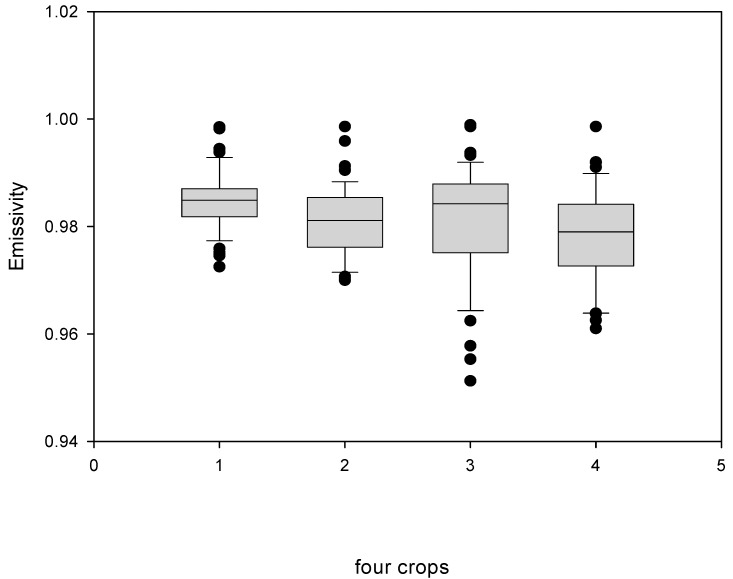
Statistical analysis of emissivity data: 1. Malabar chestnut; 2. *Paphiopedilum*; 3. *Phalaenopsis* mature; 4. *Phalaenopsis* new leaves.

The emissivity values obtained in this study were analyzed and are in Table 2. The statistical analysis of the emissivity data is shown in Figure 5.

**Table 2:** Emissivity values for the crop leaves in this study.

Crops	Mean	SD	CV
*Phalaenopsis* mature leaves	0.980943	0.010391	1.06%
*Phalaenopsis* new leaves	0.978293	0.008619	0.88%
*Paphiopedilum* leaves	0.981057	0.006641	0.68%
*Achiva macrocarpe* Malabar leaves	0.984777	0.005365	0.55%

SD: Standard deviation; CV: Coefficients of variation.

The mean emissivities were 0.9809, 0.9783, 0.981 and 0.9848 for *Phalaenopsis* mature and new leaves and *Paphiopedilum* and Malabar chestnut leaves, respectively. The mean emissivity value was higher but not significantly for *Phalaenopsis* mature leaves than new leaves. The data distribution was wider for mature than new *Phalaenopsis* leaves. Emissivity differed significantly between leaves of Malabar chestnut and the two other orchids. Malabar chestnut had the best precision measurement among the four leaf types, with no difference between *Phalaenopsis* and *Paphiopedium* leaves.

### 3.5. Emissivity for Crops in the Literature

The emissivity values of various crops from the literature are listed in Table 3. Emissivities ranged from 0.957 to 0.987. For example, the emissivity of pipe was 0.982 [29]. The emissivity for bean and tobacco were 0.957 and 0.971, respectively [15], lower than most emissivities listed in Table 3. The result may be explained by the limited performance of the infrared thermometer used in the early 1960s.

**Table 3:** Emissivity values in the literature for various plants.

Crops	Mean	sd	CV	Sources
Rape	0.98	0.01	1.02%	Rahkonen and Jakela [20]
Sow-thistle	0.98	0.01	1.02%	Rahkonen and Jakela [20]
Pine	0.982	0.009	0.92%	Arp and Phiuney [29]
Olive	0.976	0.006	0.62%	Rubio *et al.* [18]
Alfafa	0.987	0.004	0.41%	Rubio *et al.* [18]
Pipe	0.982	0.009	0.91%	Rubio *et al.* [18]
Holmoak	0.985	0.01	1.02%	Rubio *et al.* [18]
Tomato	0.980	0.01	1.02%	Lopez *et al.* [21]
Pepper	0.978	0.008	0.82%	Lopez *et al.* [21]
Cucumber	0.983	0.008	0.81%	Lopez *et al.* [21]
Courgette	0.985	0.007	0.71%	Lopez *et al.* [21]
Aubergine	0.973	0.007	0.72%	Lopez *et al.* [21]
Melon	0.978	0.006	0.61%	Lopez *et al.* [21]
Watermelon	0.981	0.009	0.91%	Lopez *et al.* [21]
Green bean	0.983	0.006	0.61%	Lopez *et al.* [21]
Red bean	0.983	0.005	0.51%	Lopez *et al.* [21]
Bean	0.957	0.005	0.52%	Fuchs and Tanner [15]
Tobacco	0.971	0.002	0.21%	Fuchs and Tanner [15]

Besides the emissivities given by Fuchs and Tanner [15], the emissivities of 16 crops in Table 3 were from 0.973 to 0.987. The emissivities of the three crops in this study were from 0.978 to 0.985, which is similar to data found in the literature (Table 3).

The CV represents the precision of the measurement. Excluding the CV values of Fuchs and Tanner [15], the CV values of the 16 crops in Table 3 ranged from 0.41% to 1.02%. The CV values in this study ranged from 0.55% (Malabar) to 1.06% (*Phalaenopsis* mature leaves), which were similar to the precision of other methods in the literature (Table 3).

The infrared thermometer used in this study is a commercial device. It is inexpensive and easy to use. By calibrating with a standard temperature environment and establishing the appropriate calibration equations, the emissivity of crop leaves is easy to determine by the method developed in this study. The emissivities were similar to those in the literature. The CV values showed that this method had good precision.

Rahknen and Jokela [20] studied three *Brassica rapa* and *Sonchus arvensi*s leaves with a reference emittance technique involving the Inframetrics 760 E imaging infrared radiometer. Lopez *et al.* [21] modified this technique and used an infrared camera to record thermographic images and determined the emissivities of nine horticultural crops. Both techniques involved delicate and expensive equipment and the methods were complicated. Many laboratories lack of this instrumentation. The method developed in this study involved use of an infrared thermometer, and the accuracy of the temperature measurement was improved with calibration equations. This technique should be simple and easy to use for most laboratories.

**Figure 6:**
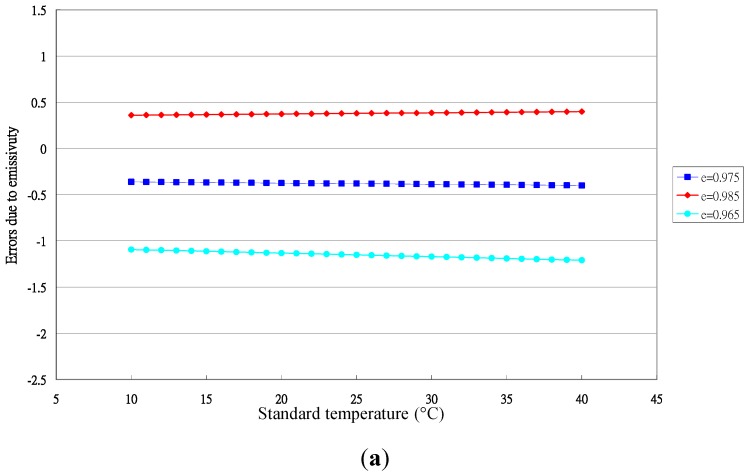

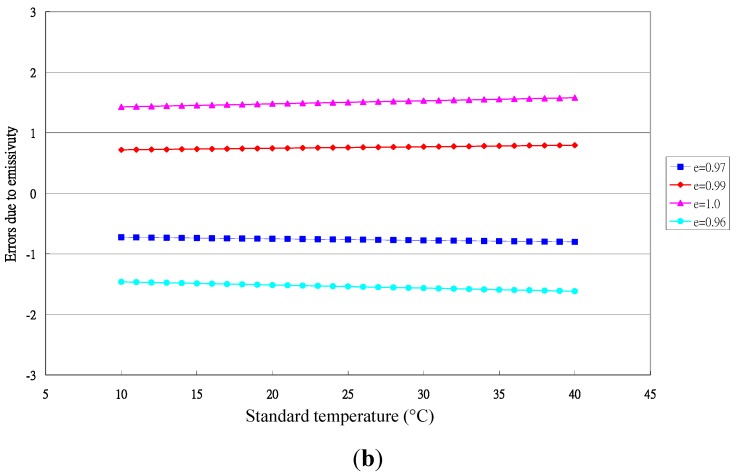
The effect of the emissivity settings on measurement errors for crop leaves. (**a**) Emissivities were assumed as 0.975, 0.985 and 0.965; (**b**) Emissivities were assumed as 0.975, 0.99, 1.0 and 0.96.

Setting a correct emissivity value for the leaf temperature measurement by infrared thermometer is key to ensuring the accuracy of the infrared thermometer. The effect of the emissivity setting on the measured errors is presented in Figure 6. If the emissivity of the target was 0.98, the errors of 0.05 deviation values calculated from Equation (12) were 0.39, −0.40 and −1.18 °C for the setting values of 0.985, 0.975 and 0.965 (Figure 6a), respectively. The errors if the deviation of the emissivity was >0.1 are presented in Figure 6b. The errors were 1.51, 0.76, −0.77 and −1.56 °C for the setting values of 0.99, 1.0, 0.97 and 0.96, respectively. Brewster [30] and Meyer *et al.* [31] suggested using the emissivity of ε=0.98 as general value for various vegetables. Lopez *et al.* [21] recommended an emissivity value of 0.98 as a reference value for measuring temperature of horticultural crops. However, some emissivities of crops, such as aubergine (ε=0.973), dried herbs (ε=0.962) and *Tridentate* L. (ε=0.97), were different from this value. The difference in emissivity for some crops may induce the errors of up to 0.5 °C, even 1.0 °C. Van Alstyne and Olson [22] mentioned that a 1 °C measurement error was found when the set emissivity was within −0.07 and 0.05 of the actual emissivity at 22 °C. The result indicated the importance of the emissivity setting for accurate temperature measurement by infrared thermometer.

Methods of emissivity determination have been mentioned by researchers [16,17,18,19,20,21,22]. These methods require a water bath or temperature controller for the reference environment and infrared thermography for temperature measurement. In this study, a simple technique was developed employing an inexpensive infrared thermometer. The emissivity of crops could be determined and the emissivity settings should be adjusted before measurement. The method developed in this study is a real-time, *in situ* technique. This method could be used for other agriculture and forestry plants.

## 4. Conclusions

In this study, the emissivities of three crop leaves were measured. The measured values were transformed into true temperatures by calibration equations to improve the measurement accuracy. The mean emissivities were 0.9809, 0.9783, 0.981 and 0.9848 for *Phalaenopsis* mature and new leaves and *Paphiopedilum* and Malabar chestnut leaves, respectively. The emissivity of Malabar chestnut leaves differed significantly from that of the two orchids. The mean emissivity was higher, but not significantly, for *Phalaenopsis* mature leaves than new leaves. The emissivities determined in this study were similar to those in the literature and the precision was similar. The major research contribution of this study was to derive a mathematical model to express the relationship between two kinds of measurement temperatures and set emissivities, and then the true emissivity can be calculated with these parameters using regression analysis. The method developed in this study is a real-time, *in situ* technique, therefore, the resulting accurate emissivity values could be used to reduce the leaf temperature measurement errors. This method could be used for other agricultural and forestry plants.
